# The identification of circular RNAs from peripheral blood mononuclear cells in systemic lupus erythematosus

**DOI:** 10.1186/s12920-021-00919-w

**Published:** 2021-03-05

**Authors:** Fengping Zheng, Xiangqi Yu, Donge Tang, Xiaoping Hong, Xinzhou Zhang, Dongzhou Liu, Yong Dai

**Affiliations:** 1 Department of Nephrology, Shenzhen People’s Hospital, The Second Clinical Medical College of Jinan University, Shenzhen, Guangdong 518020 People’s Republic of China; 2 Clinical Medical Research Center, The Second Clinical Medical College, Jinan University, Shenzhen People’s Hospital, Shenzhen, Guangdong 518020 People’s Republic of China; 3 Department of Nephrology, Yueyang First People’s Hospital, Yueyang, Hunan 414000 People’s Republic of China; 4 Department of Rheumatology and Immunology, Shenzhen People’s Hospital, The Second Clinical Medical College of Jinan University, Shenzhen, Guangdong 518020 People’s Republic of China

**Keywords:** SLE, Circular RNA, PBMCspredicted, QRT-PCR, Diagnosis

## Abstract

**Background:**

The diagnosis of systemic lupus erythematosus (SLE) is complicated. This study explores the expression of circular RNAs (circRNAs), which are closed non-coding RNAs in which the 5′ and 3′ ends are covalently linked and which work by sponging microRNAs. CircRNAs were extracted from peripheral blood mononuclear cells (PBMCs) of SLE patients to identify novel circRNA species that might be used for SLE diagnosis.

**Methods:**

Microarray was applied to screening circRNAs changes in PBMCs obtained from SLE patients (n = 10) and healthy participants (n = 10), paired for age and sex. We then verified the selected circRNAs in PBMCs using quantitative reverse transcription-polymerase chain reaction amplification (qRT-PCR) in another cohort, including ten paired SLE patients and healthy participants. The correlation between the differential circRNAs and clinical pathology of SLE were analyzed.

**Results:**

182 up-regulated and 563 significantly down-regulated circRNAs in PBMCs of patients with SLE were identified. Besides, the qRT-PCR results were consistent with the microarray results. The correlation analysis revealed that has_circRNA_100236, has_circRNA_102489, and has_circRNA_101413 were correlated with positive anti-dsDNA, thrombocytopenia, and positive IgG, respectively. Lastly, their miRNAs targets and the binding sites were predicted.

**Conclusion:**

We identified some dysregulated circRNAs in PBMCs from SLE patients, and these circRNAs may be developed as the novel biomarkers for the diagnosis of SLE.

## Background

SLE is a chronic autoimmune disease with different courses and clinical manifestations. Its etiology is highly complex, with extensive multi-systemic effects ranging from skin lesions, hematological suppression, and kidney damage to nervous system injury and musculoskeletal manifestations [1, 2]. There is growing evidence of the critical role of cytokines in SLE pathogenesis and their utility as biomarkers and targets of emerging therapies. Specifically, these immune effectors exhibit misdirection against a wide range of autoantigens, triggering signaling pathways that cause disease-specific tissue damage. The most specific laboratory targets are autoantibodies, such as anti-dsDNA and antiphospholipid antibodies. The pathogenesis and symptoms of SLE may involve the interaction of multiple factors, resulting in the frequent misdiagnosis of this disorder. Therefore, identifying new and characteristic SLE biomarkers predicting the early stage of SLE is imperative for accurate diagnosis, better symptom management, and to improve the assessment of medication efficacy in clinical trials [3].

CircRNAs comprise a large, novel class of non-coding RNAs that are highly represented in the eukaryotic transcriptome and represent naturally occurring covalently closed-loop structures wherein the 3′ and 5′ ends are interconnected [4]. The formation of circRNAs, first described in eukaryotic cells, was initially misinterpreted as the by-products of mis-splicing with no biological function [5]. Conversely, novel computational approaches have recently shown circRNAs highly stable with high abundance and exist predominantly in the cytoplasm, although they can also be harbored in exosomes [6].

To date, thousands of human circRNAs have been identified, many of which have demonstrated potential as a significant gene regulator [7]. CircRNAs have been shown to function as competing for endogenous RNAs (ceRNAs) and miRNA sponges via complementary base pairing to regulate gene expression and suppress the activity of miRNAs, respectively [8]. CircRNAs have been implicated in neurological disorders such as Alzheimer’s disease [9] as well as in pancreatic carcinoma [10] and esophageal squamous cell carcinoma [11]. ciRS-7, for example, is extensively expressed in neuroblastomas, astrocytoma, and renal cell and lung carcinomas as determined by expression analyses in various tumor cell lines [12]. Circular RNA ciRS-7, which contains over 70 miR-7 binding sites (also termed miRNA response elements; MREs), has been recently found to act as a sponge for miR-7, thus representing a miRNA inhibitor that actively suppresses miR-7 activity. Accordingly, as a by-product of their roles in gene regulation in a wide range of biological processes, the circRNAs might influence the development of many different human disorders as well [13].

In this study, we focused on characterizing the expression of circRNAs in PBMCs and identifying potential dysregulation of circRNAs expression in patients with SLE. Also, we aimed to identify specific circRNAs involved in SLE and evaluate its potential value in SLE.

## Methods

### Inclusion and exclusion criteria

Inclusion criteria: patients diagnosed with SLE and SLE disease activity index (SLEDAI) scores are equal to or greater than 5. Exclusion criteria: SLE and other immune diseases or treated with high dose (> 1 mg/kg/d) glucocorticoid and other immunosuppressants. Based on the inclusion and exclusion criteria, eight females and two male SLE patients were included in this study (Table 1). Similarly, eight females and two healthy male control were selected at the Shenzhen people’s hospital. The experimental study was approved by the Shenzhen people’s hospital ethics committee (LL-KT-2018360) and signed informed consent forms obtained from the patients.

**Table 1:** Clinical and laboratory characteristic of the study

Characteristic	SLE (n = 10)	Control (n = 10)
Age (years)	36.80 ± 9.70	33.70 ± 10.03
Sledai score	9.50 ± 3.27	N
Anti-dsDNA (±)	+	–
Thrombosis	N	N
C3 (g/l)	0.44 ± 0.23	N
C4 (g/l)	0.08 ± 0.04	N

Values presented as mean ± SD, *N* null

### The collection of samples

5 ml blood specimens/persons were collected in Ethylene Diamine Tetraacetic Acid (EDTA) anticoagulant tube. The PBMCs were isolated using Lymphoprep (Axis-Shield Diagnostics Ltd., Dundee, UK), according to the manufacturer’s protocol.

### The extraction of RNA

After collection, the fresh blood samples were placed in RNAlater (Qiagen, Hilden, Germany) immediately and keep at − 80 °C for further use. After isolated of total RNA with TRIzol (Life Technologies, Carlsbad, CA, USA), the quantity and quality of RNA were measured by using the NanoDrop ND-1000 (NanoDrop, Wilmington, DE, USA) and agarose gel electrophoresis according to the manufacturer’s protocol.

### RNA labeling

The label of RNA was implemented according to the manufacturer’s protocol (Arraystar Inc., Rockville, MD, USA). First, we removed linear RNAs by RNase R (Epicentre, Inc., Madison, WI, USA). Second, the samples were amplified and transcribed into fluorescent cRNA utilizing a random priming method (Arraystar Super RNA Labeling Kit) and purify by the RNeasy Mini Kit (Qiagen). The concentration of the labeled cRNAs (pmol Cy3/μg cRNA) were measured by using the NanoDrop ND-1000.

### Microarray hybridization

Before the microarray, the cRNA should be hybridization. In brief, 1 μl sample dilute with1μl 25 × Fragmentation Buffer and 1 μl 10 × Blocking Agent, and the mixtures were placed at constant temperature (60 °C) for half an hour. After dilution, the sample hybridized with 25 μl 2 × hybridization Buffer, and incubated in Agilent hybridization oven (Santa Clara, CA, USA) at 65 °C for up to 17 h. After hybridization, the array was performed by Axon GenePix 4000B microarray scanner (Molecular Devices, Inc., Sunnyvale, CA, USA).

### Data collection

For the data collection, genePix Pro 6.0 software (Axon) and R software package were used. The raw data extraction and grid alignment by importing scanned images were performed by genePix, and the quantile normalization of raw data and subsequent data was processed by R software. The low intensive substance was filtered and the expression data greater than 2 times background standard deviation were reserved for further analyses. To compare the profile differences between the SLE and healthy control samples, The “fold change” was conducted. To assess statistical significance, the t-test was used. The criterion of significant differential expression was set at a P-value ≤ 0.05 with a fold change ≥ 2.0. According to fold change and P-value, The analysis outputs and ranked of the differentially expressed circRNAs were filtered by using Microsoft Excel Data/Sort & Filter functionalities (Redmond, WA, USA). The volcano plot and hierarchical clustering were used to describe the results.

### Gene ontology and pathway analysis

The analysis of gene ontology (GO) [14] and Kyoto encyclopedia of genes and genomes (KEGG) pathway [15] were performed on the upregulated circRNAs and downregulated circRNAs, respectively.

### The validation of differentially expressed circRNAs by qRT-PCR

We chose the top 5 up- or down-regulated expression of circRNAs from PBMCs for results validation by qRT-PCR, respectively. The primers were designed by the circ Primers software [16] and listed in Table 2. The GAPDH served as an internal control. The parameters for the qPCR reaction were set: 95 °C for 5 min in denature step, and the annealing/extension step of 60 s at 60 °C for 40 amplification cycles. Rotor-Gene Real-Time Analysis Software 6.0 (Qiagen) was used to generate the amplification product concentration for each sample. Fold changes were analyzed by using the 2(− ∆∆Ct) method. The ratio of the sample to the internal control was calculated as the relative values of gene expression.

**Table 2:** Primers in this study

Gene name	Forward (5′–3′)	Reverse (5′–3′)
hsa_circ_101260	CGCCCCATTTGTACACCATG	GCAGGCTCCCAACCATGATA
hsa_circ_101413	CTACGACCCACAGCAGAAGA	GCTTTAGTTTCTCGGGCAGG
hsa_circ_102489	GAACAAGCCATCCACTCCAG	TTCACTTCCAAGCACAGCAC
hsa_circ_103906	AAAGGGGATGAAGCCTGGAA	CTCCACTCAAGGTCCTCACT
hsa_circ_100263	GACTACCGTTCCCTTGAGCT	GTCCTGATCTCCCGGTGTAA
hsa_circ_104081	ACTCTTCACTGCCATTGGTT	TCTTTCTTACCCCACAACCCA
hsa_circ_103421	CATTGTGAGTGTGCTAGCCC	GCTTCCACTTCTCTCCTCGT
hsa_circ_101269	ACGGGAAGAAGCATGGGTTA	ACTGTGAGTCTTTGGCCAGT
hsa_circ_104102	CCGGAATTCAAAACCTGGCA	CACGGTTCTCAACATCAGCA
hsa_circ_101705	AGAATCTCACTCTGCTGCCC	TGGTGAATGGTGGATGGTTTG
GAPDH	ATGGGGAAGGTGAAGGTCG	GGGGTCATTGATGGCAACA

### Data analysis and annotation of circRNAs/miRNA interaction

The Arraystar miRNA target prediction software (TargetScan & Miranda) was usd to predict the circRNA/miRNA interactions, and the circRNAs/miRNA interaction information was annotated in detail. Also, the sequences of the MREs were analyzed.

### Statistical analysis

Statistical analyses were performed after libraries were established. The circRNAs were mapped to the genome and a summary was produced for the known circRNAs alignment. Cluster analysis and differential expression were performed for the circRNAs. qRT-PCR results were shown as mean ± SD, T-tests were applied to compare the expression level. The clinicopathologic features were compared using Pearson’s chi-squared test or Fisher’s exact test.

## Results

### Quality control

To ensure successful microarray analysis, all quality criteria established by the manufacturer should be satisfied. First, the quality and quantity of RNA from blood sample was measured by Nanodrop and agarose gel electrophoresis. The results showed that the A260 /A280 ratio value was closed to 2.0 (ratios between 1.8 and 2.1 were acceptable), and the A260/A230 ratio was greater than 1.8. Additionally, the staining of the ribosomal RNA bands of 28S and 18S were sharp and intensely, in contrast, the tRNA and 5S ribosomal RNA were presented as thin bands, meaning that the prepared RNA was of high purity, without degradation, and without contamination by DNA, protein, or other impurities, and the sample was qualified (data not shown).

### Identification of differentially expressed circRNAs in SLE

**Fig. 1:**
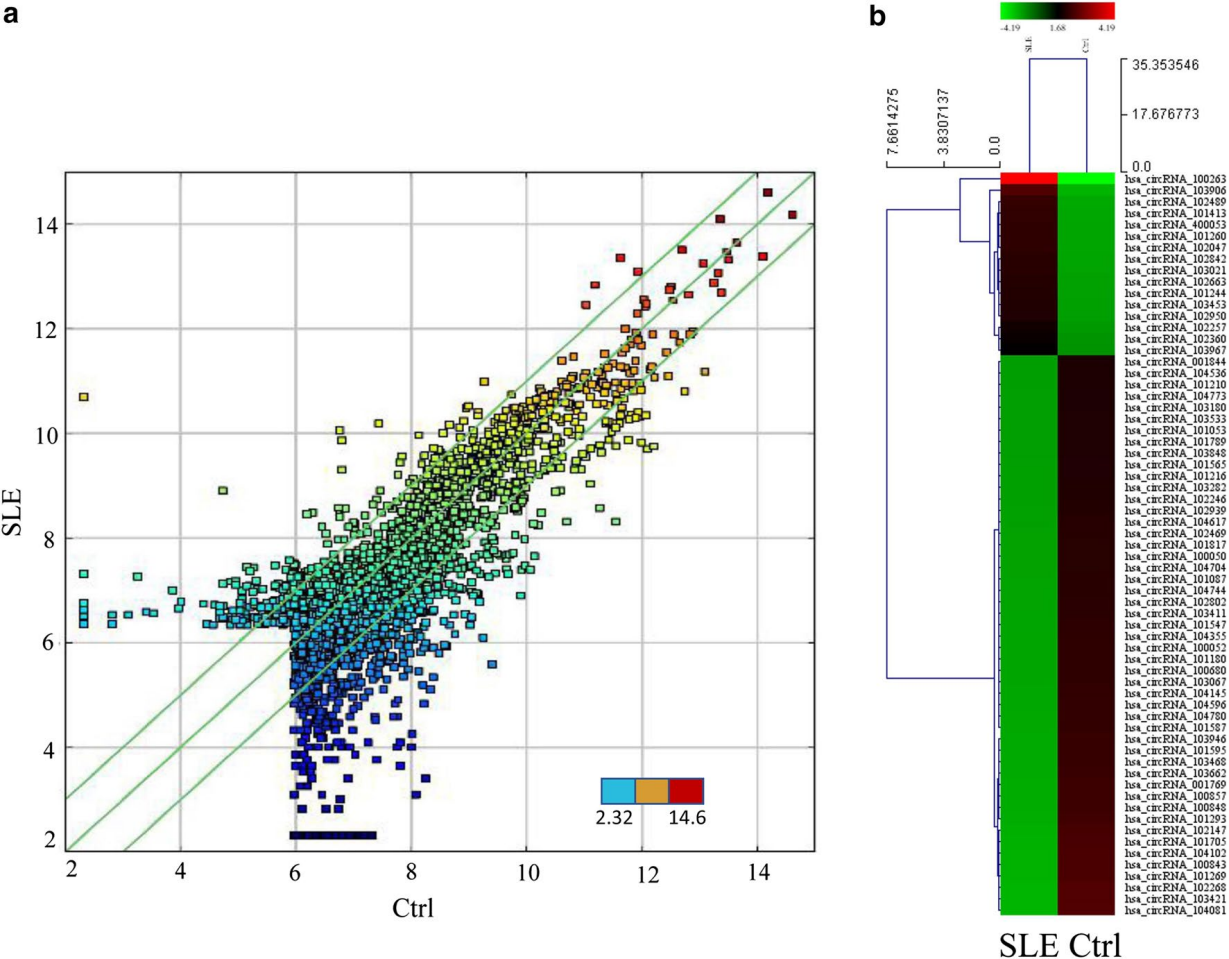
Differential circRNAs in PBMCs of SLE. A Scatterplot of circRNA expression variation and reproducibility between SLE and control samples. The values of X and Y axes in the scatterplot represent the normalized signal values of the samples (log2 scaled). The dots represent the expression level from low (blue, 2.32) to high (red, 14.6). The green lines illustrate the fold change. The circRNAs above the top green line and below the bottom green line exhibit > 2.0-fold change between SLE and controls. B Hierarchical clustering to distinguish differentially expressed circRNAs between SLE and control samples. Each column represents one sample, and each row represents one circRNA. The dendrogram shows the relationships among the sample expression levels

We identified 745 circRNAs that exhibited significant differences (fold change ≥ 2.0, *p* < 0.05) in expression in PBMC between patients with SLE and controls, among which 182 and 563 were up- and down-regulated (Fig. 1a). Also, hierarchical clustering, which arranges samples into groups based on their expression levels and allows the generation of hypotheses regarding the relationships among samples, was performed based on all target circRNAs (Fig. 1b). The figure demonstrated precise circRNAs expression profiles between the SLE and healthy control groups.

### The potential biological function of the differential circRNAs in SLE

**Fig. 2:**
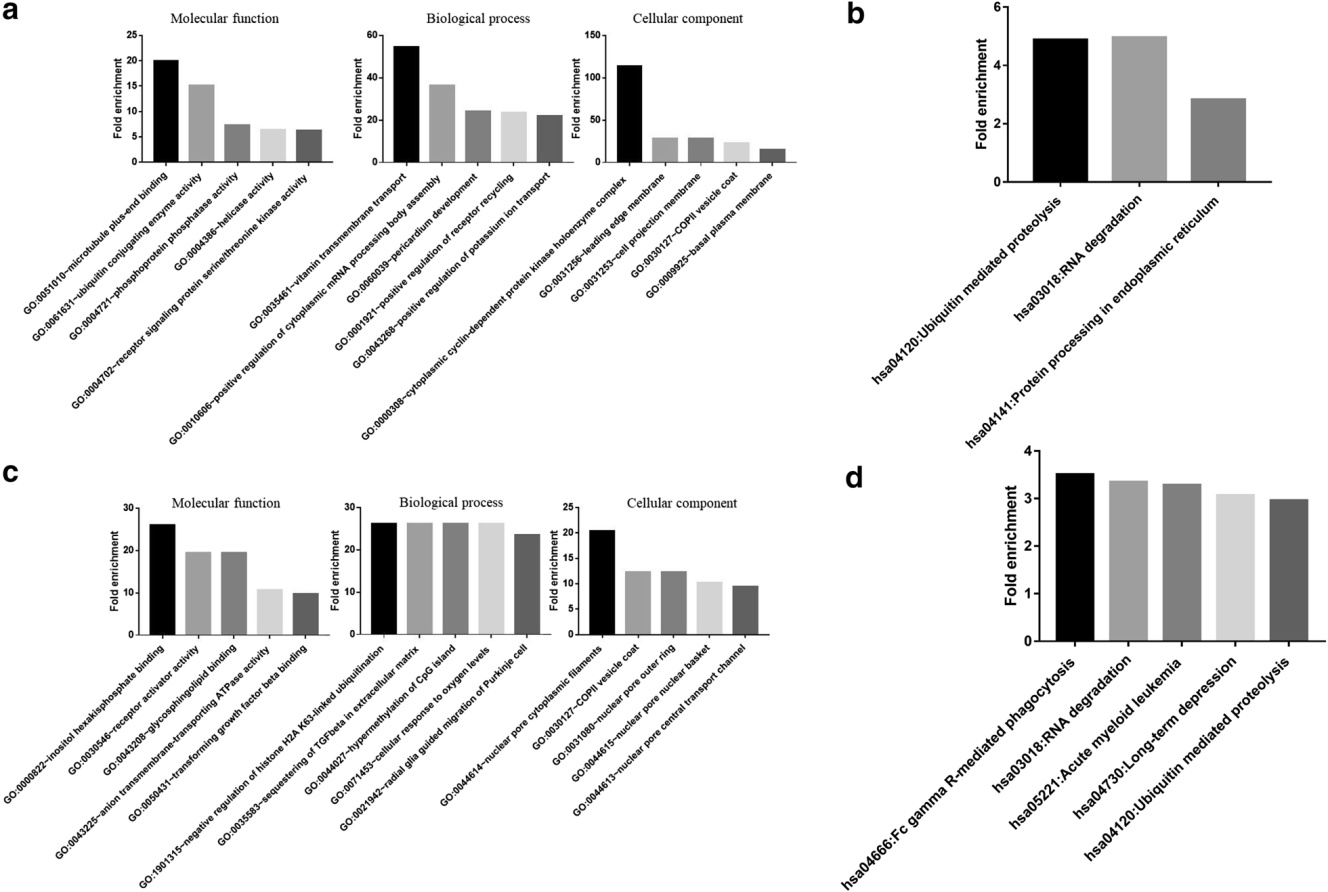
GO and KEGG pathway. **a** The top five-fold-enrichment molecular function, biological process, and cellular component of upregulated circRNAs. **b** The pathway of upregulated circRNAs involved. **c** The five-fold-enrichment molecular function, biological process, and cellular component of the down-regulated circRNAs. D The pathway of downregulated circRNAs involved

To evaluate the potential biological function of the differential circRNAs in SLE, the Gene Ontology and KEGG pathway analyses were performed. The gene ontology (molecular function, biological process, and cellular component) and the KEGG pathway of upregulated (Fig. 2a, b) and downregulated (Fig. 2c, d) circRNAs in SLE were analyzed separately. The biological process of upregulated circRNAs showed that they play a positive role in regulating cytoplasmic mRNA processing body assembly, which was consistent with its miRNA sponge role. The biological process of down-regulated circRNAs showed that they are involved sequestering of transforming growth factor beta in extracellular matrix, and the level of transforming growth factor beta were low in SLE patients with active disease [17], suggesting that transforming growth factor beta may be involved in the pathogenesis of SLE. Collectively, the results showed that the upregulated gene and downregulated genes involved various biological processes. Further investigation should be needed to clarify the exact roles of the upregulated circRNAs and downregulated circRNAs in SLE.

### qRT-PCR validation

**Fig. 3:**
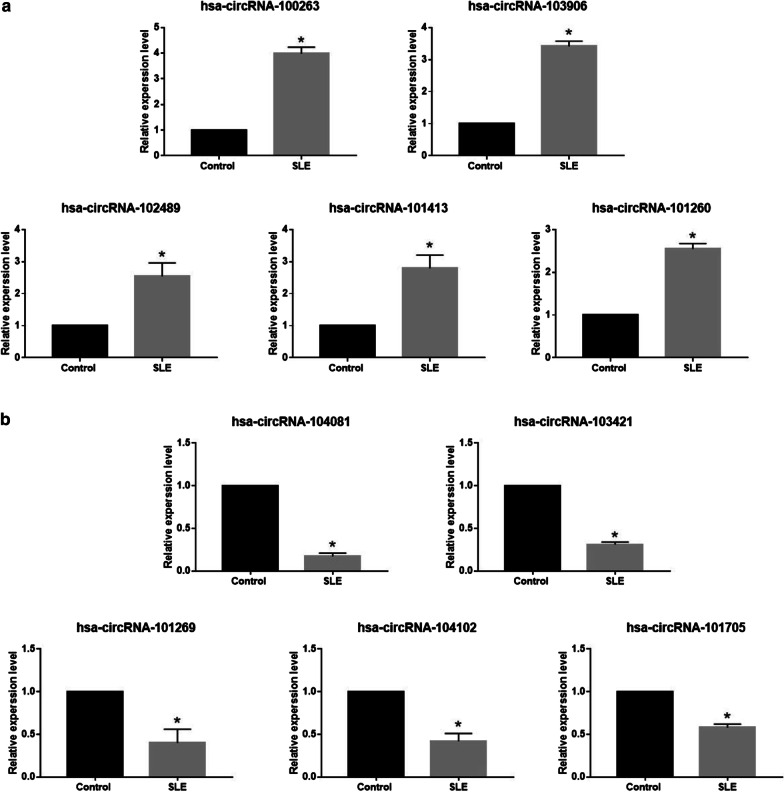
Real-time quantity PCR verified ten circular RNAs expression inPBMCs. **a** Upregulated circRNAs in PBMCs of SLE. **b** Downregulated circRNAs in PBMCs of SLE. qRT-PCR results were shown as mean ± SD, T-test applied to compare the expression level

To verify our observations, we confirmed the expression of the top 5 up-regulated circRNAs (100263, 103906, 102489, 101413, 101260) and the top 5 down-regulated circRNAs (104081, 103421, 101269, 104102, 101705) in the PBMCs by qRT-PCR. GAPDH used as internal control. Specifically, the relative direction of expression changes between SLE and control groups, as measured by qRT-PCR for the tested circRNAs was consistent with that identified by the original array expression analysis. The results above and calculated ratios of gene expression shown in Fig. 3.

### The correlation of circRNAs and clinical pathologies in SLE

To determine the correlation of circRNAs and clinical pathologies in SLE, we analyzed the correlation between clinicopathologic characteristics and the top five up-regulate circRNAs expression (Table 3). The results revealed that has_circRNA_100236, has_circRNA_102489, and has_circRNA_101413 were correlated with positive anti-dsDNA, thrombocytopenia and positive IgG, respectively, suggested that these circRNAs may be developed as a diagnostic marker for SLE.

**Table 3:** The correlation between circRNAs and clinicopathologic characteristics in SLE

SLE criterion	has-circRNA (fold change > 2.5 means high)														
	100263		*P*	103906		*P*	102489		*P*	101413		*P*	101260		*P*
	H	L		H	L		H	L		H	L		H	L	
Malar rash (+)	8	1	0.73	8	1	0.73	6	3	0.49	5	4	0.389	3	6	0.2
Malar rash (−)	1	0		1	0		1	0		1	0		1	0	
Photosenstivity (+)	2	1	0.11	3	0	0.49	3	0	0.292	1	2	0.26	1	2	0.78
Photosenstivity (−)	7	0		6	1		4	3		5	2		3	4	
Discoid rash (+)	2	0	0.6	2	0	0.6	2	0	0.301	2	0	0.197	1	1	0.75
Discoid rash (−)	7	1		7	1		5	3		4	4		3	5	
Oral uclers (+)	1	0	0.73	1	0	0.73	0	1	0.107	1	0	0.389	0	1	0.39
Oral uclers (−)	8	1		8	1		7	2		5	4		4	5	
Arthritis Anti-MPO (+)	2	0	0.6	2	0	0.6	1	1	0.533	1	1	0.747	1	1	0.75
Anti-MPO (−)	7	1		7	1		6	2		5	3		3	5	
Serositis	N.A	N.A	N.A	N.A	N.A	N.A	N.A	N.A	N.A	N.A	N.A	N.A	N.A	N.A	N.A
Renal disorder	2	1	0.11	3	0	0.49	1	1	0.183	2	1	0.667	1	2	0.78
≥ 500 versus < 500	7	0		6	1		6	2		4	3		3	4	
Neurologic disoder	N.A	N.A	N.A	N.A	N.A	N.A	N.A	N.A	N.A	N.A	N.A	N.A	N.A	N.A	N.A
Hematologica disoder															
Leukopenia (+)	3	0	0.49	2	1	0.11	3	0	0.175	1	2	0.26	1	2	0.78
Leukopenia (−)	6	1		7	0		4	3		5	2		3	4	
Lymphopenia (+)	3	0	0.49	3	0	0.49	3	0	0.175	2	1	0.778	2	1	0.26
Lymphopenia (−)	6	1		6	1		4	3		4	3		2	5	
Thrombocytopenia (+)	2	0	0.6	2	0	0.6	0	2	0.016*	1	1	0.747	0	2	0.2
Thrombocytopenia (−)	7	1		7	1		7	1		5	3		4	4	
ANA															
Anti-dsDNA (+)	9	0	0.02*	8	1	0.73	6	0	0.49	5	4	0.389	3	6	0.2
Anti-dsDNA (−)	0	1		1	0		1	3		1	0		1	0	
Anti-Smd1 (+)	2	0	0.6	2	0	0.6	1	1	0.533	1	1	0.747	1	1	0.75
Anti-Smd1 (−)	7	1		7	1		6	2		5	3		3	5	
Immunologic disoders															
IgG (+)	6	0	0.2	5	1	0.39	3	3	0.2	2	4	0.035*	1	5	0.07
IgG (−)	3	1		4	0		4	0		4	0		3	1	
IgM (+)	1	0	0.73	1	0	0.73	1	0	0.49	5	0	0.389	1	0	0.2
IgM (−)	8	1		8	1		6	3		1	4		3	6	
Anti-C3 (+)	6	1	0.49	6	1	0.49	5	2	0.88	4	3	0.778	2	5	0.26
Anti-C3 (−)	3	0		3	0		2	1		2	1		2	1	
Anti-C4 (+)	5	1	0.39	5	1	0.39	4	2	0.778	4	2	0.598	2	4	0.6
Anti-C4 (−)	4	0		4	0		3	1		2	2		2	2	
SLEDAI			0.89			0.78			0.85			0.82			0.8

SLEDAI P means Pearson coefficient, indicated high expression of circRNAs positive related to SLEDAI

Next, we analyzed the correlation between the three circRNAs (has_circRNA_100236, has_circRNA_102489, has_circRNA_101413) and SLEDAI scores. The results showed that the expressions of three circRNAs (has_circRNA_100236, has_circRNA_102489, has_circRNA_101413) were positively correlated with SLEDAI score, suggested that these circRNAs could be developed as novel biomarkers for the diagnosis of SLE (Table 3). The confirmation in a larger cohort is needed.

### The interaction between differential expression of circRNAs and miRNAs

**Fig. 4:**
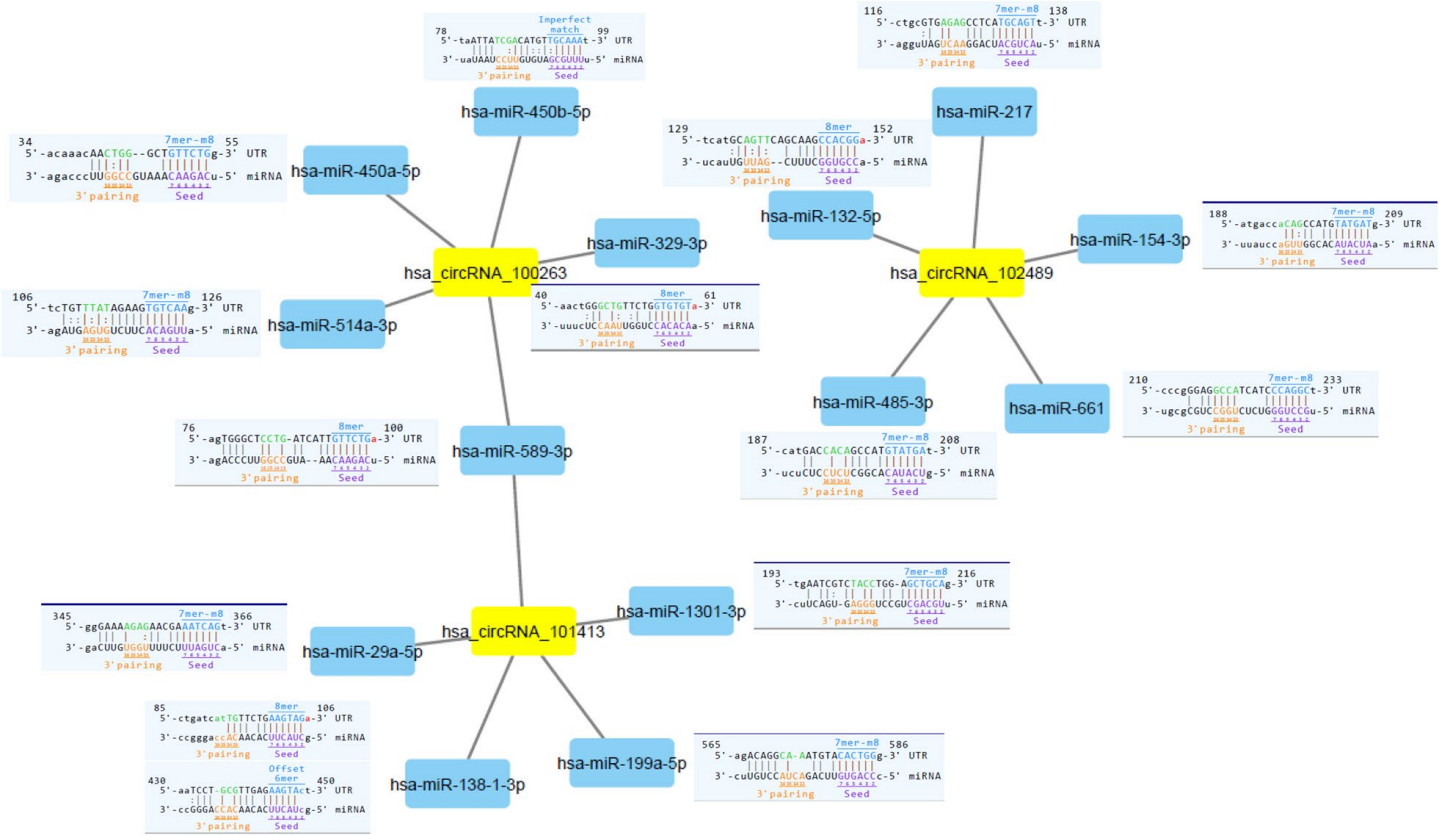
Detailed Annotation for circRNA-miRNA interactions. Representative notation example is showing the complementary situation of has_circRNA_100236, has_circRNA_102489, has_circRNA_101413, and their target miRNAs and also the predicted sponge site

CircRNAs exerted its function by sponge miRNA. In the current study, we determined the sequence overlap (potential MREs) between circRNA and miRNA differentially expressed in SLE using miRNA target prediction software (Arraystar). In this study, the differentially expressed circRNAs were notated in detail. For example, in hsa_circRNA_101413, the 360th–365th nucleotides are homologous to the 5′ terminal region in the highly expressed gene GPATCH2L and represent a complete match to the seed region 7mer-m8 (positions 2–8) of miR-29a-5p. We showed the circRNAs with diagnostic value and their miRNAs targets and the predicted binding site in Fig. 4.

## Discussion

CircRNAs were expressed widely and variedly in numerous types of human cells and play a crucial role in regulating gene expression, acting as miRNAs sponges and ceRNAs, circRNAs chelate miRNAs via their MREs, thus removing the inhibitory effects of miRNAs and augmenting the appearance of the respective target genes. Accordingly, the sequences of circRNAs are highly conserved, and their expression is stable in mammalian cells [18]. Although circRNAs discovered decades ago, it becomes a critical research topic only in recent years. Although alterations in circRNAs have been reported to associate with the development and progression of numerous human diseases [19], the profile of circRNAs in patients with SLE has not been previously described.

In this study, we identified 745 differentially expressed circRNAs in PBMCs of patients with SLE, among which 182 significantly up-regulated and 563 were significantly down-regulated. The GO and KEGG pathway analysis results indicated that these circRNAs might participate in SLE's pathogenesis through various processes and pathways. And we verified the microarray results by qRT-PCR, and the results showed that our sequencing results were convinced. The circ Primer is software for us to better understanding the structure of circRNAs and designed the specific primers for circRNAs study. Next, we explored the clinical significance of the top five upregulated circRNAs. The results suggested that has_circRNA_100236, has_circRNA_102489, and has_circRNA_101413 could be developed as novel diagnostic biomarkers for SLE because they were correlated with positive anti-dsDNA, thrombocytopenia and positive IgG, respectively, and also associated with SLEDAI score positively. A larger cohort of SLE patients should be included to validate the results.

To date, the exact role of these circRNAs in the pathogenesis of SLE remains mostly unknown. Regarding the role of circRNAs, it has been widely reported as a miRNAs sponge. So, we predicted its MREs by Target-scan and miRnada, aiming to elucidate their roles in the SLE. circRNAs acted as miRNAs sponge to regulate gene expression to affect diseases. Almost every differentially expressed circRNA identified in our study has its corresponding miRNA binding sites.

Based on the computer algorithm, the target of circRNAs and the binding site were predicted. Three clinical significance circRNAs and their target miRNAs were shown here. Experiments should verify the binding site because it was based on computer algorism.

It was well known that miRNAs were involved in the pathogenesis of SLE. In particular, miR-30a and miR-1246 contribute to the control of B cell hyperactivity through the silencing of Lyn and EBF1, respectively. Accordingly, their expression dysregulation in B cells might associate with SLE pathogenesis [20]. miR-29b was significantly up-regulated in the naive B cell subset of SLE compared to that of healthy controls, whereas miR-29c and miR-let-7d and MiR-let-7e were significantly down-regulated [21]. miR-513-5p was recently identified and abnormally expressed in SLE T cells compared to healthy controls. In turn, it has demonstrated that the induction of miR-217 led to the activation of Akt in glomerular mesangial cells by TGF-beta, suggesting an association between mesangial cell activation or kidney disorder and miRNAs [22]. Furthermore, significant up-regulation of miR-148a in patients with SLE and lupus-prone mice compared to healthy controls was found in CD4+T cells. Subsequent investigations showed that miR-148a suppressed DNMT1 expression by targeting a protein-coding region of the transcript.

Conversely, significantly lower expression of miRNA 17-5p was found in patients with SLE, especially in those not receiving treatment [23]. This study also observed the expression pattern of miRNA 17-5p and identified its target gene, E2F1. miRNA 17-5p was further demonstrated to influence the cell cycle by promoting the G1/S transition by targeting a considerable protein network, resulting in cells' proliferation in patients with SLE [23]. miR-29b overexpression in the cells of patients with SLE led to the reduction of sp1 and DNMT1 expression and resulted in DNA hypomethylation in the CD4+T cells of healthy controls as well as in the increased expression of genes encoding CD11a and CD70. Similarly, MiR-148a directly binds to the 3′-UTR region of the DNA methyltransferase DNMT1 transcript, inhibiting DNMT1 expression in SLE T cells [24].

Furthermore, miR-130b-3p has emerged as a biomarker for patients with early-stage lupus nephritis (LN) because of the up-regulation of its level in serum [25]. Association analysis has shown that overexpression of miR-130b-3p is linked to the renal chronicity index and 24-h proteinuria and serum triglyceride levels in patients with early-stage LN and the epithelial-mesenchymal transition [25]. CD4+T cells contribute to the reduction of miR-125a. miR-125a down-regulation, as observed in our study, was shown to be positively correlated with Kruppel-like factor 13 (KLF13), which was associated with the expression of RANTES in T cells [26]. Thus, miR-125a may play a part in lupus CD4+T cells by targeting the 3′-UTR of KLF13.

This study has shown that differentially expressed circRNAs and their corresponding MREs in SLE should raise deep concern to many because of its significance for diagnosing SLE.

However, whether these circRNAs could develop as a clinical diagnosis and precise therapy need further investigation. Also, some shortages occurred in this study. First of all, the sample size in our study is not big enough. Secondly, the data we presented in this study is a description of circRNAs and miRNAs we found in SLE, and the interactions between circRNAs and miRNAs predicted by the software should be further verified in the benchwork.

## Conclusion

In conclusion, in this study, we described differentially expressed circRNAs and their corresponding MREs in SLE. Our findings should draw enough attention, and it should further be explored. Maybe those differentially expressed circRNAs we found to play an essential role in SLE's occurrence and development.

## Data Availability

The datasets generated and/or analysed during the current study are available in the ArrayExpress repository, the accession is E-MTAB-10125, available at: https://www.ebi.ac.uk/arrayexpress/experiments/E-MTAB-10125/.

## Abbreviations

SLE: Systemic lupus erythematosus
CircRNAs: Circular RNAs
PBMCs: Peripheral blood mononuclear cells
qRT-PCR: Quantitative reverse transcription-polymerase chain reaction amplification
SLEDAI: SLE disease activity index
GO: Gene ontology
KEGG: Kyoto encyclopedia of genes and genomes
